# Magnitude and Timing of Leaf Damage Affect Seed Production in a Natural Population of *Arabidopsis thaliana* (Brassicaceae)

**DOI:** 10.1371/journal.pone.0030015

**Published:** 2012-01-19

**Authors:** Reiko Akiyama, Jon Ågren

**Affiliations:** Plant Ecology and Evolution, Department of Ecology and Genetics, Evolutionary Biology Centre, Uppsala University, Uppsala, Sweden; Netherlands Institute of Ecology, The Netherlands

## Abstract

**Background:**

The effect of herbivory on plant fitness varies widely. Understanding the causes of this variation is of considerable interest because of its implications for plant population dynamics and trait evolution. We experimentally defoliated the annual herb *Arabidopsis thaliana* in a natural population in Sweden to test the hypotheses that (a) plant fitness decreases with increasing damage, (b) tolerance to defoliation is lower before flowering than during flowering, and (c) defoliation before flowering reduces number of seeds more strongly than defoliation during flowering, but the opposite is true for effects on seed size.

**Methodology/Principal Findings:**

In a first experiment, between 0 and 75% of the leaf area was removed in May from plants that flowered or were about to start flowering. In a second experiment, 0, 25%, or 50% of the leaf area was removed from plants on one of two occasions, in mid April when plants were either in the vegetative rosette or bolting stage, or in mid May when plants were flowering. In the first experiment, seed production was negatively related to leaf area removed, and at the highest damage level, also mean seed size was reduced. In the second experiment, removal of 50% of the leaf area reduced seed production by 60% among plants defoliated early in the season at the vegetative rosettes, and by 22% among plants defoliated early in the season at the bolting stage, but did not reduce seed output of plants defoliated one month later. No seasonal shift in the effect of defoliation on seed size was detected.

**Conclusions/Significance:**

The results show that leaf damage may reduce the fitness of *A. thaliana*, and suggest that in this population leaf herbivores feeding on plants before flowering should exert stronger selection on defence traits than those feeding on plants during flowering, given similar damage levels.

## Introduction

Herbivory can reduce plant fitness and thereby influence both population dynamics and selection on defense traits, e.g. [1]–[5]. However, tolerance to damage, quantified by the slope of the relationship between damage and plant fitness [6], may vary considerably, and determining how plant characteristics and environmental conditions influence the fitness consequences of herbivory in natural plant populations remains a major challenge, e.g. [7]–[11].

Tolerance to leaf damage should depend on the timing of damage and several hypotheses have been formulated to predict how tolerance changes seasonally. Some emphasize the importance of time available for recovery from damage and suggest that herbivory early in the season and early during development should be easier to compensate than leaf damage late in the season and during reproduction [7], [8]. Other hypotheses suggest that changes in tolerance reflect differences in available resources and the extent to which plant fitness is limited by photosynthate relative to other resources [2], [12]–[14]. Following this reasoning, it has been predicted that tolerance to leaf herbivory in annual plants should increase from the seedling stage until flowering as a result of resource accumulation before flowering [12], [14]. When evaluating seasonal changes in the effects of leaf damage on plant fitness, it may thus be important to consider not only the timing of damage but also the life-history stage at which plants are defoliated.

The timing of herbivory should affect the magnitude of the response, but it may also influence which fitness components are affected. In annual plants, flower production is typically determined earlier than seed size, and mean seed mass is therefore predicted to be more sensitive to damage occurring late in the season than are number of flowers and number of fruits [15]. There is some support for the predictions that leaf damage early in the season is more detrimental than damage late in the season, e.g. [13], [15], [16], and that the components of fitness most affected by herbivory shift along ontogeny in annual plants [17]. However, because responses to herbivory may often be context-dependent [7], [9], [11] and most studies of seasonal changes in tolerance to leaf herbivory have been conducted in the greenhouse rather than in the field, e.g. [15], [16] additional studies of the effects of herbivory on plant performance in natural populations are needed.

In this study, we conducted two experiments in a natural population of the annual herb *Arabidopsis thaliana* to determine the fitness consequences of leaf damage of different severity before and after flowering. Like most populations of *A. thaliana* in the native range [18]–[20], the study population has a winter-annual life cycle. Seeds germinate in late summer and in the autumn, and plants overwinter as vegetative rosettes and develop through vegetative, bolting, and flowering stages the following spring. In the study population, flowering typically begins in late April or early May, and fruits mature in late June. In a first experiment, between 0 and 75% of the leaf area was removed in May from plants that flowered or were about to start flowering. In a second experiment, 0, 25%, or 50% of the leaf area was removed from plants on one of two occasions (in mid April when plants were either in the vegetative rosette or bolting stage, or in mid May when plants were flowering). We tested the predictions that (a) plant fitness decreases with increasing damage, (b) tolerance to defoliation is lower before flowering than during flowering, and (c) defoliation before flowering reduces number of seeds more strongly than defoliation during flowering does, but the opposite is true for effects on seed size. In addition, we documented the seasonal pattern of damage in the study population to determine whether seasonal changes in tolerance to damage were correlated with differences in risk of herbivory. This study fills an important gap in the literature by contributing much needed information on the ecology of natural populations of the plant model species *A .thaliana* in its native range.

## Results

### Plant Fitness and Seed Size vs. Magnitude of Damage

**Figure 1:**
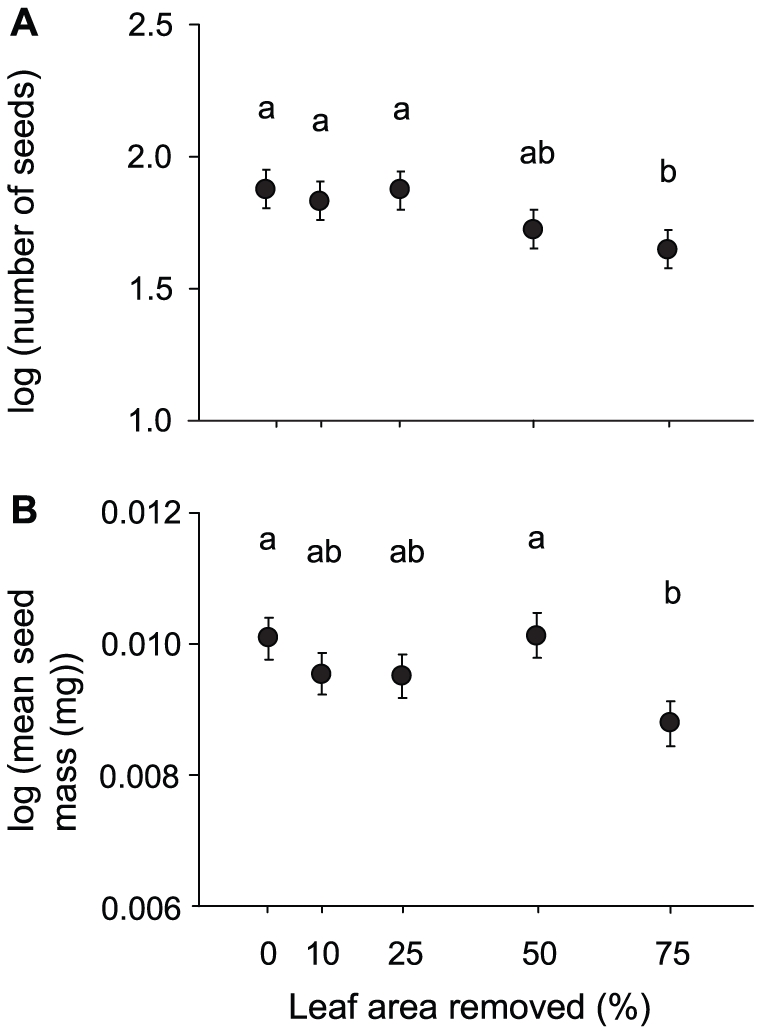
Effects of defoliation on the number and size of seeds. (A) log (number of seeds) and (B) log (mean seed mass [mg]) of *Arabidopsis thaliana* (least-square means ± S.E). Different letters indicate statistically significant differences in means (*P*<0.05) based on Tukey's HSD test. Linear regression of treatment least-square means on proportion of leaf area removed: log (number of seeds), y = −0.003×+1.891, *P*<0.05, R^2^ = 0.88, log (mean seed mass [mg]), y = −0.0000105×+0.0000083, *P* = 0.29, R^2^ = 0.35.

In the first experiment, both overall fitness in terms of the number of seeds produced per experimental plant and seed size were affected by defoliation and tended to decrease with increasing proportion of leaf area removed (Table 1, Fig. 1). Removal of 50% of the leaf area reduced seed production by 29% (back-transformed least-square mean, control 76 seeds, 50% defoliation 54 seeds). The effect of defoliation on plant fitness was mainly a function of its influence on fecundity of survivors. All plants in the control, 10% defoliation and 25% defoliation treatments survived to reproduction, while 93% of the plants survived in the 50% and 75% defoliation treatments. Mean seed mass was significantly reduced only after the most severe defoliation treatment (Fig. 1B). The number of seeds produced was positively related to rosette area at the time of the defoliation treatment (Table 1).

**Table 1:** Effects of defoliation (0, 10%, 25%, 50%, or 75% of leaf area removed; fixed effect), block (random effect), rosette size, and proportion of leaf area removed by herbivores before the defoliation treatment on the number of seeds and mean seed mass of *Arabidopsis thaliana* in the first experiment analyzed with mixed-model ANOVA.

Source	Number of seeds	Mean seed mass
	*F* or *χ* ^2^	*F* or *χ* ^2^
Defoliation	2.7*	3.0*
Block	14.3***	4.3*
Rosette area at treatment	16.2***	0.7
Damage before treatment	0.8	0.5

The response variables were log-transformed prior to analysis. *F* is given for all independent variables except block, for which *χ* ^2^ is given.

*P<0.05,

***P<0.001.

### Seasonal Shift in Tolerance to Damage

**Figure 2:**
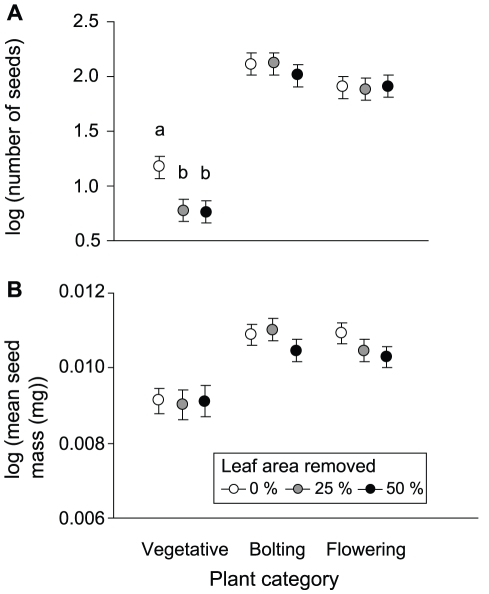
Effects of defoliation and plant category on the number and size of seeds. (A) log (number of seeds) and (B) log (mean seed mass [mg]) of *Arabidopsis thaliana* (least-square means ± S.E.). Different letters indicate statistically significant differences in means based on Tukey's HSD test performed separately by plant category (vegetative rosette defoliated early in the season, bolting plant defoliated early in the season, or flowering plant defoliated a month later).

In the second experiment, the effect of defoliation on plant fitness varied among plant categories (damaged early in the season at the vegetative rosette stage, damaged early in the season at the bolting stage, damaged one month later at the flowering stage) as indicated by a significant defoliation × plant category interaction (Table 2, Fig. 2A). Removal of 50% of the leaf area in mid April reduced number of seeds produced per plant by 60% among plants that were vegetative rosettes and by 22% among plants that had reached the bolting stage at the time of damage (back-transformed least-square mean, vegetative rosette, control 15 seeds, 50% defoliation 6 seeds; bolting, control 129 seeds, 50% defoliation 100 seeds). Damage of the same magnitude to flowering plants in mid May did not reduce seed production (flowering, control 79 seeds, 50% defoliation 81 seeds). Analyses conducted separately by cohort indicated that the reduction in fitness was statistically significant among plants defoliated at the vegetative rosette stage, but not among plants defoliated at the bolting stage (Table 3, Fig. 2A).

**Table 2:** Effects of defoliation (0, 25% or 50% of leaf area removed), plant category (vegetative rosette defoliated early in the season, bolting plant defoliated early in the season, or flowering plant defoliated a month later; fixed effects), block (random effect), and proportion of leaf area removed by herbivores before the defoliation treatment on the number of seeds and mean seed mass of *Arabidopsis thaliana* in the second experiment analyzed with mixed-model ANOVA.

Source	Number of seeds	Mean seed mass
	*F* or *χ* ^2^	*F* or *χ* ^2^
Defoliation	3.4*	1.1
Plant category	164.6***	22.2***
Defoliation × plant category	2.5*	0.8
Block	60.4***	3.5*
Damage before treatment	1.0	0.1

The response variables were log-transformed prior to analysis. *F* is given for all independent variables except block, for which *χ* ^2^ is given.

*P<0.05,

***P<0.001.

**Table 3:** The effects of defoliation (fixed effect), block (random effect), and proportion of leaf area removed by herbivores before the defoliation treatment on the number of seeds of *Arabidopsis thaliana* in the second experiment analyzed with mixed models separately by plant category (vegetative rosette defoliated early in the season, bolting plant defoliated early in the season, or flowering plant defoliated a month later).

Source	Vegetative	Bolting	Flowering
	*F* or *χ* ^2^	*F* or *χ* ^2^	*F* or *χ* ^2^
Defoliation	6.4*	0.5	0.2
Block	18.4**	6.1*	40.0***
Damage before treatment	1.1	2.6	3.0

The response variables were log-transformed prior to analysis. *F* is given for all independent variables except block, for which *χ* ^2^ is given.

*P<0.05,

**P<0.01,

***P<0.001.

Defoliation of vegetative plants in mid April tended to reduce both survival and fecundity of surviving plants, but neither effect was statistically significant. Among plants defoliated at the vegetative stage, survival was 0.71 in the control, 0.54 in the 25% defoliation, and 0.56 in the 50% defoliation treatment (effect of defoliation in logistic regression, *χ* ^2^ = 2.7, df = 2, *P* = 0.25). Back-transformed least-square mean number of seeds produced by reproducing plants was 40 seeds in the control, 28 seeds in the 25% defoliation treatment, 31 seeds in the 50% defoliation treatment (effect of defoliation in ANOVA, *F*_2,41.6_ = 2.6, *P* = 0.09).

Plants that were at the vegetative stage at the time of the early defoliation had lower fitness compared to the other two plant categories (Fig. 2A). Both survival and fecundity were lower among plants defoliated at the vegetative stage early in the season compared to plants defoliated at the bolting stage on the same occasion and plants defoliated at the flowering stage a month later (survival, effect of plant category in logistic regression, *χ* ^2^ = 77.0, df = 2, *P*<0.0001; fecundity, effect of plant category in ANOVA, *F*_2,236_ = 124.7, *P*<0.0001).

In the second experiment, no statistically significant effect of defoliation on mean seed mass was recorded, but plants defoliated at the vegetative stage early in the season produced smaller seeds compared to the other two plant categories (Table 2, Fig. 2B).

### Seasonal Variation in Herbivory

Leaf herbivory did not differ markedly between life-history stages, was of similar magnitude in the two years of study, and did not vary much through the season. In 2006, the proportion of plants with leaf damage was 22% on May 19. The proportion of the leaf area removed by herbivores ranged from 1% to 40%, with a mean of 8% (Table 4). At the time of the damage census, 70% of the plants were in the bolting stage, while the rest had begun flowering. No difference in leaf damage was detected between bolting and flowering plants (Wilcoxon rank sum test, *P* = 0.65). In 2007, the proportion of plants damaged by leaf-feeding herbivores was similar to that in 2006 and ranged from 24% to 26% at censuses from 15 April to 27 May (Table 4). The proportion of the leaf area removed by herbivores from damaged plants varied widely (range 1%–80%), but the mean proportion was relatively low (9% at the first census and about 3% at the remaining censuses; Table 4). The stage structure of the population changed gradually over the season. At the first census, 42% of the population was in the vegetative rosette stage and the rest was bolting (*N* = 214), while at the final census 88% of the plants had reached the flowering stage and 10% was bolting (*N* = 183). At censuses when a sufficient number of plants in different stages made comparisons meaningful, no difference in the proportion of leaf area damaged was recorded between vegetative and bolting plants (15 April, Wilcoxon, *P* = 0.21), between vegetative, bolting or flowering plants (29 April, Kruskal-Wallis, *P* = 0.24) or between bolting and flowering plants (13 May, Wilcoxon, *P* = 0.90).

**Table 4:** Stage structure, proportion damaged plants, and leaf area removed (%) from damaged plants in the *Arabidopsis thaliana* study population on May 19, 2006, and on four different dates in 2007.

Day	Proportion of plants			*N*	Proportion damaged	Leaf area removed	
	Vegetative rosette	Bolting	Flowering			Mean	Range
*2006*							
May 19	0	0.70	0.30	200	0.22	7.6%	1%–40%
*2007*							
April 15	0.42	0.58	0	214	0.25	8.9%	1%–80%
April 29	0.19	0.60	0.21	214	0.25	3.1%	1%–15%
May 13	0.05	0.30	0.65	195	0.26	3.1%	1%–15%
May 27	0.02	0.10	0.88	183	0.24	3.5%	1%–20%

## Discussion

The present study has demonstrated that both the extent and timing of leaf damage influence the fitness of *Arabidopsis thaliana* under field conditions. The detrimental effects of defoliation on the number of seeds produced and seed size tended to increase with increasing damage, and defoliation of vegetative plants early in the season reduced seed production more strongly than did defoliation of bolting plants at the same time and defoliation of flowering plants a month later. Like many other winter annuals, most *A. thaliana* populations complete the life cycle in spring or early summer, and may thereby avoid herbivory from several potential insect herbivores [21]. However, this and previous studies [21], [22] demonstrate that herbivory can still be substantial in the native range of *A. thaliana* and vary widely among individual plants.

### Seasonal Change in Tolerance to Damage

Seasonal changes in risk of herbivory and resource availability may influence costs and benefits of tolerance and resistance to herbivory and thereby select for ontogenetic shifts in tolerance [2], [16]. In the second experiment, tolerance to defoliation was lower among plants defoliated at the vegetative rosette stage early in the season than among bolting plants defoliated at the same time, and among flowering plants defoliated a month later. Removal of 50% of the leaf area from vegetative rosettes early in the season reduced seed production by 60%, while similar damage to bolting plants early in the season reduced seed production by 22%, and had no effect when applied to flowering plants a month later . This suggests that the plants defoliated at the vegetative rosette stage were more limited by photosynthates at the time of damage than the other plant categories, cf. [11]. Differences among stages in tolerance to damage were not correlated with seasonal shifts in the risk of herbivory in the natural population. The proportion of plants that showed evidence of damage from herbivores was rather constant across the season and no statistically significant differences in herbivore damage between life-history stages were recorded. If anything, the proportion of leaf area removed from damaged plants tended to be higher at the first census compared to censuses conducted later in the season in 2007. Instead, the seasonal shift in tolerance may be related to changes in resource status, cf. [2], [9]. Plants may be particularly vulnerable to damage early in the season because their stored resources are limited and leaf damage may affect the production of new leaves and meristems in the rosette, and thus future productivity. Finally, because physiological response elicited by tissue damage may vary across ontogeny [23] and because the production of induced defences may often be costly [24], [25], seasonal shifts in tolerance may also reflect changes in the strength or costs associated with damage-induced defence reactions. Several factors may thus have contributed to the documented seasonal increase in tolerance to damage, and additional studies are required to determine the relative importance of resource availability *per se* and of differences in the strength and consequences of hormonal responses triggered by damage.

A seasonal increase in tolerance to damage may result in an associated reduction in selection for resistance against herbivory. Consistent with this prediction, an ontogenetic increase in tolerance has been shown to be associated with a decrease in leaf glucosinolate concentration in the annual herb *Raphanus sativus* [16]. In *A. thaliana*, glucosinolate concentration in the leaf rosette [26] and the biosynthesis of some defence-related proteins in response to methyl jasmonate [27] but see [28], have similarly been found to decrease from the vegetative rosette to flowering stage. To reveal the factors shaping selection on ontogenetic patterns of tolerance and resistance in natural *A. thaliana* populations, future studies should quantify seasonal variation in costs and benefits of tolerance and resistance traits in relation to risk of damage from specific herbivores and pathogens.

The experimental design of the present study does not allow the effects of ontogenetic stage and plant vigor to be distinguished fully. At the time of the early defoliation, vegetative and bolting plants differed in developmental stage, but also in size, and these differences were associated with a difference in number of seeds produced at the end of the season (cf. difference in fitness of control plants Fig. 2). The documented difference in response to defoliation may thus reflect an increase in tolerance to defoliation from the vegetative rosette stage to the reproductive stage, a difference in tolerance between plants of different vigor, or a combination thereof.

In this study, we used scissors to create different levels of leaf damage. Mechanical damage does not mimic all aspects of herbivore damage. In particular, herbivory can induce defence reactions that are not triggered by mechanical damage [29]–[31]. Because the production of induced defences may often be associated with a cost [24], [25], the present experiment may have underestimated the fitness consequences of leaf removal by herbivores. However, studies of other plant species indicate that differences between mechanical damage and damage caused by herbivores are less prevalent when effects on growth and reproduction are examined than when effects on metabolism and resistance to secondary herbivory are considered [32].

Support for the hypothesis that tolerance to defoliation in annuals increases from the vegetative rosette stage to the reproductive stage has previously been obtained in greenhouse experiments with *A. thaliana* [33] but see [28], *Plantago aristata* [17], *Raphanus sativus* [16], *Sesbania macrocarpa* and *S. vesicaria* [15], and in a field study of *Ipomoea purpurea* [13]. In contrast, no evidence of an ontogenetic increase in tolerance to defoliation was observed in a greenhouse experiment with *Senecio vulgaris* [34], or in a field study of *Cucurbita pepo* [35]. Several factors may have contributed to the variable outcomes including differences in experimental design, in the exact developmental stages examined in combination with non-linear changes in tolerance, e.g., [10], and differences in fitness components considered.

In contrast to the present study and a greenhouse study of three accessions of *A. thaliana* [33], Barto and Cipollini [28] did not detect lower tolerance to damage before than during flowering in *A. thaliana*. The difference in result is not likely to be related to differences in developmental stages included for study because in all three studies vegetative plants with on average six true leaves and flowering plants that had recently begun to flower were defoliated. However, the three studies differ in several other ways that may have influenced their results. First, different defoliation methods were employed. In the present study, all leaves were damaged with scissors to a predefined degree in the defoliation treatments, in the study conducted by Tucker and Avila-Sakar [33] larvae of *Trichoplusia ni* were allowed to feed on plants to a given level of damage was achieved, while in Barto and Cipollini [28] either the 50% youngest or the 50% oldest leaves were removed completely. The fitness consequences of a given level of defoliation may depend on whether damage is evenly distributed among all leaves or not [36], and it is conceivable that the pattern of defoliation also influences the importance of the timing of defoliation. Second, while we focused on seasonal changes in effects of defoliation on plant fitness, the previous two studies focused on ontogenetic shifts in tolerance to damage. We defoliated plants on two occasions, early in the season and in the middle of the *A. thaliana* growing season, whereas in the other two studies, plants were damaged as they reached a particular developmental stage. As a consequence, the difference in ontogenetic stage between plants defoliated at the vegetative rosette stage and at the bolting stage was confounded by a difference in vigor in the present study, while in the previous studies effects of ontogenetic stage on response to damage were confounded by possible effects of day of defoliation. Further experiments are needed to determine the importance of this difference. Finally, the response of different populations was examined. Natural populations of *A. thaliana* differ in resistance to insect herbivores [37], in the composition of glucosinolates [38], and in response to apical meristem damage [39]. Moreover, recent studies indicate that the degree to which tolerance to leaf damage [33], glucosinolate activation [23], and response to methyl jasmonate [27] changes throughout ontogeny may also vary among populations. Taken together, it is clear that additional studies are required to determine the importance of environmental conditions and experimental procedures for the detection of ontogenetic shifts in tolerance to defoliation in annual plants, and also to explore the extent to which this characteristic varies genetically among and within natural populations.

### Effect of Damage to Flowering Plants

The strongest detrimental effect of leaf damage was recorded among plants defoliated at the vegetative stage early in the season, but defoliation during flowering also reduced the number and size of seeds produced in the first experiment (Fig. 1, Table 1). A comparable effect size was detected for bolting plants in the second experiment, although the reduction of seed production following defoliation was not statistically significant in this experiment. Defoliation during flowering may reduce seed production both because defoliation reduces the amount of nutrients that can be reallocated from leaves to developing seeds and because defoliation should reduce carbon gain of remaining leaves. Nitrogen is reallocated from leaves to reproductive structures after the completion of leaf expansion in *A. thaliana* [40], and reduced nutrient availability could limit seed production. Reduced leaf area may also decrease photosynthesis. However, this effect may be less important as carbon gain of reproductive *A. thaliana* to a large extent can be attributed to photosynthesis of inflorescences [41].

### Effect of Damage on the Number and Size of Seeds

The results of the present study only partly supported the hypothesis that fitness components that respond to damage shift along ontogeny from the number of seeds to seed mass. The reduction in seed production caused by defoliation in mid April was larger than the reduction following defoliation a month later, but the effect of defoliation on seed mass did not increase seasonally. Similarly, in a growth-room experiment with the annual herb *Plantago aristata*, defoliation before flowering reduced the number of seeds more strongly than defoliation during flowering and fruiting, while no such ontogenetic shift in the negative effect of defoliation on mean seed mass was observed [17]. In contrast, little evidence of shifts in the relative magnitude of effects on different components of reproduction was detected in a greenhouse study of *Sesbania macrocarpa* and *S. vesicaria* [15]. Taken together, seasonal changes in the relative importance of effects of defoliation on different components of reproductive output have not been detected in all species studied, and when they have been observed, they have not involved changes in the magnitude of effects on all individual components. While early defoliation may reduce the number of seeds more strongly than late defoliation in many species, the negative effect of defoliation on seed mass appears less variable among life-history stages.

### Conclusions

The present study has shown that leaf damage may significantly reduce the fitness of *A. thaliana* and suggests that in this population, leaf herbivores feeding on plants early in the season should exert stronger selection on defense traits than leaf herbivores feeding on plants at later in the season, given similar damage levels. The fitness consequences of herbivory critically depend on the seasonal timing of damage also in perennial plant species, e.g. [10], [42]–[44]. There is thus ample evidence that a comprehensive understanding of the effects of leaf herbivory on the numerical dynamics and evolutionary trajectories of natural plant populations requires that both the timing and magnitude of damage are considered.

## Materials and Methods

### Study Species and Study Site

*Arabidopsis thaliana* (L.) Heynh. (Brassicaceae) is a self-compatible annual herb, which is native to Eurasia [45]. It has a wide latitudinal range, from 68°N in northern Scandinavia to 0° [20], and is typically found in disturbed habitats [46]. The species is subject to damage from slugs and snails feeding on leaves [21] and from insect herbivores feeding on leaves [47], [48] and fruits [22].

The study was conducted in an *A. thaliana* population at Rödåsen (62°48′N,18°12′E) in the High Coast area of the province Ångermanland, central Sweden. The population is located in dry meadow vegetation on a steep slope facing south-east, approximately 175 m above the sea level. In the study population, herbivore damage is mainly caused by insect herbivores, including *Plutella xylostella*, and gastropods feeding on leaves in spring (J. Ågren, personal observation). The proportion of plants damaged by folivores varies among years, but may often be in the order of 20–25% or higher. Completely defoliated plants have been observed already soon after snow melt in April (R. Akiyama and J. Ågren, personal observation).

### Field Experiments

To examine how plant survival and fecundity (number of seeds produced by reproducing plants), and seed size are affected by the magnitude of defoliation and by the timing of defoliation, we performed two experiments. In the first experiment, conducted in 2006, plants were marked in groups of five, and within groups ( = blocks) individual plants were randomly allocated to one of five treatments: 0% (control), 10%, 25%, 50%, or 75% of the area of each rosette leaf removed with scissors (*N* = 30 per treatment). Damage was inflicted on 20 May, when most plants were about to start flowering (some had begun flowering). Prior to defoliation, the number of leaves per plant, rosette diameter, and natural damage (scored as the proportion of leaf area removed) were recorded. Rosette diameter was determined with a digital calliper to the nearest mm, and proportion of leaf area removed by herbivores was estimated by eye to the nearest 1% when 10% or less of the leaf area had been removed and to the nearest 5% for plants that had lost more than 10% of their leaf area. At the time of the treatment, the plants had produced 9.1±2.7 leaves (mean ± SD), and had a rosette area (estimated as π [rosette diameter/2]^2^) of 59.8±34.0 mm^2^ (*N* = 150). Natural herbivory was low. Twenty-three percent of the plants had leaves damaged by insect herbivores (*N* = 150), and among damaged plants the mean proportion of leaf area removed was 6.3±5.0% (median [range], 5% [1–20%], *N* = 35). Plant size and proportion of leaf area removed by herbivores did not vary among treatments (one-way ANOVA, number of leaves, *F*_4,145_ = 0.24, *P* = 0.91; rosette area, *F*_4,145_ = 1.67, *P* = 0.16; Kruskal-Wallis test, leaf damage, *P* = 0.91).

In the second experiment, conducted in 2007, we defoliated plants early in spring before flowering (18 April) or during flowering (19 May). On 18 April, approximately 60% of the plants in the population had reached the bolting stage, while on 19 May, the great majority of plants had begun flowering. For this experiment, we therefore identified three plant categories: plants defoliated early in spring at the vegetative rosette stage, plants defoliated early in spring at the bolting stage, and plants defoliated a month later at the flowering stage. For each category, 40 triplets of plants were selected one to three days before the experimental defoliation. Triplets were arranged in blocks, with one triplet of each plant category forming a block. Each plant within a triplet was randomly assigned to one of three defoliation treatments: 0% (control), 25%, and 50% of the area of each rosette leaf removed with scissors. The number of leaves, rosette diameter, and proportion of the leaf area removed by herbivores were recorded on 17 April for plants defoliated in mid April, and on 16 May for plants defoliated a month later. Rosette area was estimated as described above. Plants defoliated early in the season at the vegetative rosette stage had fewer leaves and smaller rosettes than those defoliated early in the season at the bolting stage, and those defoliated a month later at the flowering stage (mean ± SD, number of leaves, vegetative rosette 6.2±1.1, bolting 9.3±1.8, flowering 6.9±1.9; rosette area, vegetative rosette 40.3±14.9 mm^2^; bolting 103.5±36.7 mm^2^; flowering 89.6±51.2 mm^2^), but the number of leaves and rosette area did not differ between defoliation treatments (two-way ANOVA, number of leaves, plant category *F*_2,351_ = 120.2, *P*<0.0001, defoliation *F*_2,351_ = 0.3, *P* = 0.77, plant category × defoliation *F*_4,351_ = 0.3, *P* = 0.90; rosette area, plant category *F*_2,351_ = 93.2, *P*<0.0001, defoliation *F*_2,351_ = 0.04, *P* = 0.96, plant category × defoliation *F*_4,351_ = 0.1, *P* = 0.99). The proportion of plants damaged by herbivores was lower among plants defoliated at the vegetative rosette stage (8%, *N* = 120) than among plants defoliated at the bolting and flowering stages (27% and 26%, respectively), but did not differ between defoliation treatments (logistic regression, likelihood ratio tests, plant category χ^2^ = 17.8, *P* = 0.0001, defoliation χ^2^ = 0.9, *P* = 0.63, plant category × defoliation χ^2^ = 3.3, *P* = 0.51). Damage levels were typically low (mean damage among damaged plants, 3.4±3.4%, median [range], 2% [1–20%], *N* = 75), and did not differ significantly between plant category or defoliation treatments (two-way ANOVA, arcsine square-root transformed proportion of leaf area removed, plant category *F*_2,65_ = 1.6, *P* = 0.22, defoliation *F*_2,65_ = 0.2, *P* = 0.80, plant category × defoliation *F*_4,65_ = 0.8, *P* = 0.53).

In both experiments, plant fitness was quantified as number of seeds produced (coded as zero for plants that did not survive to reproduce), and we examined treatment effects both on this measure of overall fitness and on its two components, survival to reproduction and fecundity of survivors. Because seed size has been found to be correlated positively with seedling survival in *A. thaliana* [49] and other species [50], we also quantified effects on mean seed mass. Survival until fruit maturation (0 or 1) and the number of fruits produced by reproducing plants were recorded at fruit maturation. For each reproducing plant, we estimated the mean number of seeds per fruit by counting the number of seeds produced by up to five fruits. To estimate total number of seeds produced, the mean number of seeds per fruit was multiplied with the number of fruits. For each reproducing plant, mean seed mass was obtained by determining the mass of up to 60 seeds.

### Variation in Herbivory among Developmental Stages and across the Season

To obtain quantitative data on leaf herbivory on a larger number of plants and to characterize the seasonal pattern of herbivory in the study population, we recorded damage to two additional sets of plants. In 2006, we recorded developmental stage and leaf damage to 200 plants randomly selected across the population on May 19. In 2007, we randomly selected and marked 214 plants across the population in mid April. The developmental stage and leaf damage to these plants were recorded on 15 April, 29 April, 13 May, and 27 May. In both years, we quantified leaf damage following the same procedure as in the defoliation experiment.

### Statistical Analyses

Statistical analyses were conducted using SAS version 9.2 and JMP version 5.0.1. We used logistic regression in JMP to examine the effect of defoliation on survival. Because survival was very high (range 93%–100%) except among plants defoliated at the vegetative stage in the second experiment, the effect of the defoliation treatment on survival was examined separately by developmental stage. We used mixed models to assess the effects of defoliation treatment (fixed effect), block (random effect), rosette area at defoliation, and proportion of leaf area removed by herbivores before the defoliation treatment on number of seeds produced by survivors (fecundity) and by all plants (overall fitness), and on mean seed mass in the first experiment (PROC MIXED in SAS). Initial analyses also included the defoliation × rosette area interaction, but since this interaction was never statistically significant, it was excluded from the final models. In the analysis of the second experiment, we included defoliation treatment, plant category (vegetative rosette defoliated early in the season, bolting plant defoliated early in the season, or flowering plant defoliated a month later), the defoliation × plant category interaction (fixed effects), block (random effect), and proportion of leaf area removed by herbivores before the defoliation treatment as explanatory variables in the models. Number of seeds per plant and mean seed mass were log-transformed prior to analysis to improve normality of residuals. When significant main effects were detected, post-hoc tests were carried out using Tukey's HSD test. To determine whether seed production and mean seed mass decreased with increasing damage in the first experiment, we regressed least-square treatment means on proportion of leaf area experimentally removed using PROC REG in SAS. We tested the significance of the random effect in the mixed models using the log-likelihood ratio statistic [51]. We compared the −2 residual log likelihood of the full model with that of the model without the random factor. The significance of the difference in the residual log likelihood was determined by comparing it with a chi-square value twice the nominal significance level with one degree of freedom [51].
